# Cholera Rapid Diagnostic Tests for the Detection of *Vibrio cholerae* O1: An Updated Meta-Analysis

**DOI:** 10.3390/diagnostics11112095

**Published:** 2021-11-13

**Authors:** Basilua Andre Muzembo, Kei Kitahara, Ayumu Ohno, Anusuya Debnath, Keinosuke Okamoto, Shin-Ichi Miyoshi

**Affiliations:** 1 Graduate School of Medicine, Dentistry and Pharmaceutical Sciences, Okayama University, Okayama 700-8530, Japan; keikitahara@okayama-u.ac.jp (K.K.); py386nyz@okayama-u.ac.jp (A.O.); anusuyadebnath@yahoo.co.in (A.D.); okamot-k@okayama-u.ac.jp (K.O.); miyos-s@okayama-u.ac.jp (S.-I.M.); 2 Collaborative Research Center, Okayama University for Infectious Diseases in India, Kolkata 700010, India

**Keywords:** rapid test, cholera, *Vibrio cholera* O1, sensitivity, specificity, accuracy, update

## Abstract

The rapid diagnosis of cholera contributes to adequate outbreak management. This meta-analysis assesses the diagnostic accuracy of cholera rapid tests (RDTs) to detect *Vibrio cholerae* O1. Methods: Systematic review and meta-analysis. We searched four databases (Medline, EMBASE, Google Scholar, and Web of Science up to 8 September 2021) for studies that evaluated cholera RDTs for the detection of *V. cholerae* O1 compared with either stool culture or polymerase chain reaction (PCR). We assessed the studies’ quality using the QUADAS-2 criteria. In addition, in this update, GRADE approach was used to rate the overall certainty of the evidence. We performed a bivariate random-effects meta-analysis to calculate the pooled sensitivity and specificity of cholera RDTs. Results: Overall, 20 studies were included in this meta-analysis. Studies were from Africa (*n* = 11), Asia (*n* = 7), and America (Haiti; *n* = 2). They evaluated eight RDTs (Crystal VC-O1, Crystal VC, Cholkit, Institut Pasteur cholera dipstick, SD Bioline, Artron, Cholera Smart O1, and Smart II Cholera O1). Using direct specimen testing, sensitivity and specificity of RDTs were 90% (95% CI, 86 to 93) and 86% (95% CI, 81 to 90), respectively. Cholera Sensitivity was higher in studies conducted in Africa [92% (95% CI, 89 to 94)] compared with Asia [82% (95% CI, 77 to 87)]. However, specificity [83% (95% CI, 71 to 91)] was lower in Africa compared with Asia [90% (95% CI, 84 to 94)]. GRADE quality of evidence was estimated as moderate. Conclusions: Against culture or PCR, current cholera RDTs have moderate sensitivity and specificity for detecting *Vibrio cholerae* O1.

## 1. Introduction

Despite centuries of effort, cholera (an acute diarrheal disease caused by *Vibrio cholerae* O1 or O139) remains a high-volume health issue, especially in Africa and the Indian subcontinents [1]. Channels of cholera transmission include the ingestion of food or drinking water contaminated with feces from an infected person or direct contact with infected feces. The risk of cholera outbreak is high in underprivileged communities with rudimentary access to safe water, adequate sanitation, and hygiene (WaSH) [2]. There were seven instances of cholera pandemics during the 19th and 20th centuries. Six of them emerged from the Ganges Delta in the Indian subcontinent and one from Indonesia (the ongoing seventh pandemic). From there, this human pathogen has spread rapidly across other continents such as Africa, the Americas, Europe, and other parts of Asia, killing millions of people [1,3]. 

The disease remains a killer. About 95,000 deaths (range: 21,000–143,000) are reported every year worldwide [1]. Without any treatment, patients with severe cholera can die of dehydration and hypovolemic shock within hours after the onset of symptoms. Fortunately, timely treatment may limit cholera-related fatality, i.e., with appropriate case management, death would occur in <1% of cholera patients [4]. Laboratory testing using microbiological culture and/or polymerase chain reaction (PCR) is required to confirm the etiology of cholera for strong public health responses. However, in some settings where cholera usually thrives, special laboratory equipment or trained laboratory technicians might not be readily available. Fortunately, cholera rapid diagnostic tests (RDTs) are used to screen patients with suspected cholera, and yield qualitative results within 30 min [5]. They prove especially useful in remote settings where microbiological culture and molecular testing are not easily accessible.

The Global Task Force on Cholera Control (GTFCC) recommends that cholera RDTs should have a sensitivity and a specificity of at least of 90% and 85%, respectively [6]. In our previous systematic review and meta-analysis [7], we reported that the current cholera RDTs have suboptimal pooled sensitivity (91%) and specificity (80%).

Some of the limitations of our previous meta-analysis [7] are the non-assessment of the overall quality of evidence, lack of comparative data of RDTs performance across diverse geographical regions, and inclusion of RDTs that had positive or negative readings for *V. cholera O139*. In the interim, field studies including novel brands cholera RDT (Crystal VC-O1 and Smart II) have become available. Therefore, an updated synthesis of the accuracy of cholera RDTs is needed to assist clinicians and the global public health community to grasp a thorough picture of current cholera RDTs accuracy.

In this context, we aim to provide an updated summary of the accuracy of the current cholera RDTs and address some of the limitations of our previous meta-analysis.

## 2. Methods

We carried out a systematic review and meta-analysis of studies that evaluated the performance of the current RDTs in detecting *V. cholerae* O1 in stool samples compared to either stool culture or PCR. The Preferred Reporting Items for a Systematic Review and Meta-Analysis of Diagnostic Test Accuracy Studies (PRISMA-DTA) were followed [8]. This review is registered with the International Prospective Register of Systematic Reviews (PROSPERO CRD42021233124). 

### 2.1. Data Sources and Searches

The search methods used were the same as those in our previous meta-analysis [7]. We searched MEDLINE through PubMed, EMBASE, Google Scholar, and Web of Science for studies published up to 8 September 2021, with no restrictions on language. We also checked and viewed the references of the included studies. (More details on the search strategy and study selection are available in our previously published meta-analysis [7].) In brief, eligible studies included cross-sectional studies with a sample size of at least 20 specimens. Studies were excluded if they used case-control designs, studies reporting only analytical sensitivity and specificity, and review articles.

### 2.2. Outcomes

In this updated meta-analysis, our primary outcome was the overall pooled sensitivity and specificity of RDTs to identify *V. cholerae* O1.

*V. cholerae* O139 test line readings were excluded. 

### 2.3. Data Extraction and Quality Assessment

Two investigators (B.A.M. and K.K.) independently screened citations (titles and abstracts). 

They also abstracted data and assessed the quality of the studies (risk of bias and applicability concerns) using the Quality Assessment of Diagnostic Accuracy Studies 2 (QUADAS-2) tool [9]. In addition, in this analysis, the Grading of Recommendations Assessment, Development and Evaluation (GRADE) approach was used to rate the quality of evidence for sensitivity and specificity [10]. 

Before beginning data extraction, we designed a data-extraction form. Extracted data included raw data on: the true positive; false positive; false negative; and true negative. This was used to construct 2 × 2 tables for applicable conditions. Any disagreements were resolved through consensus. 

### 2.4. Data Analysis

Methods used here are as described in our previous meta-analysis [7]. We used the Stata software (version 16, StataCorp LP, College Station, TX, USA) to analyze data and generating plots. We constructed 2 × 2 tables of test results to calculate: the pooled sensitivity; specificity; positive likelihood ratio (LR; i.e., the ratio of individuals with the disease who test positive, to those who test positive but do not have the disease) and negative LR (i.e., the ratio of individuals with the disease who test negative, to those who test negative and do not have the disease); and the diagnostic odds ratio (DOR; i.e., the ratio of the odds of positivity when the disease is present to the odds of positivity in the non-diseased) with 95% confidence intervals (CIs). 

Meta-analysis was carried out using the generalized linear mixed model of the bivariate random effects model to account for the frequent heterogeneity expected from meta-analysis of diagnostic test accuracy studies [11]. 

Heterogeneity across studies was assessed by visual inspection of the shape of the hierarchical summary receiver-operating characteristic (HSROC) curves [12]. We did not use the *I^2^* statistic to assess heterogeneity. Other potential sources of heterogeneity were assessed during sensitivity analyses.

Data from studies that had evaluated more than one index test with the same specimens were all considered as data points and included in the analyses. In sensitivity analysis, studies were stratified into three geographic regions (i.e., Africa, Asia, and the Americas). We also performed a sensitivity analysis on Crystal VC because it had sufficient data points to be pooled separately. All results are presented with their 95% CIs in parenthesis. 

## 3. Results

### 3.1. Literature Search

Our updated search resulted in 7957 unique studies (Supplementary Figure S1), of which 3 met our inclusion criteria [13,14,15], yielding 8 new data points. These new data points were added to 37 data points from 17 studies from our previous meta-analysis [7]. Therefore, in this updated meta-analysis, we included 20 studies [13,14,15,16,17,18,19,20,21,22,23,24,25,26,27,28,29,30,31,32] with a total of 45 data points. 

### 3.2. Characteristics of Included Studies

Table 1 describes the 20 included studies. The details of these 20 studies are shown in Table A1 in Appendix A. These studies evaluated eight RDT brands. Of these, Crystal VC was the most frequently studied RDT (15 studies). Other index tests included: Cholkit and Institut Pasteur cholera dipstick (three studies each); SD Bioline (two studies); Artron (one study); Smart (one study); Smart II Cholera O1(one study); and Crystal VC-O1 (one study). Crystal VC-O1 [13] and Smart II Cholera O1 [14] are the newest tests not included in our previous meta-analysis [7]. 

The studies were conducted in fourteen countries: four in Bangladesh [17,18,22,29]; three in India [14,15,26]; two in Haiti [23,31]; and one each in Cameroon [20]; Democratic Republic of the Congo [25]; Guinea Bissau [27]; Kenya [13]; Malawi [16]; Mozambique [28]; Nigeria [21]; South Sudan [32]; Tanzania [24]; Uganda [30]; and Zambia [19]. The three new studies included in this updated meta-analysis were conducted in Kenya [13] and India [14,15].

Seven studies from Africa [16,19,21,24,25,27,32] and one from Haiti [23] stated clearly that RDTs were evaluated during outbreaks.

These studies provided 45 data points (with 19,280 stool specimens). 32 out of 45 used direct stool testing (with 15,877 stool specimens) and 13 used alkaline peptone water (APW) enrichment before testing. One study [14] that evaluated various RDTs with two different gold standard tests contributed to three additional data points using PCR as the gold standard (Table A2 in Appendix A).

An overview of the methodological assessment of included studies is summarized in Table A3 in Appendix A. As in our previous meta-analysis [7], for the patient selection domain, high or unclear risk of bias was the main concern. We judged that the risk of bias was high or unclear in more than half of the studies (11/20; 55%), mostly related to patients’ unclear inclusion or exclusion criteria. Most studies used conventional culture methods as a reference standard. However, PCR was also performed to confirm the etiologic agent in ten studies [13,14,18,19,20,22,25,27,29,32]. In these studies, PCR results were not always congruent with the results of conventional culture methods. Three studies [20,27,32] used PCR alone as the reference standard and three other studies [13,14,25] combined both PCR and culture as the reference standards.

### 3.3. Meta-Analysis

#### 3.3.1. Overall Performance

**Figure 1:**
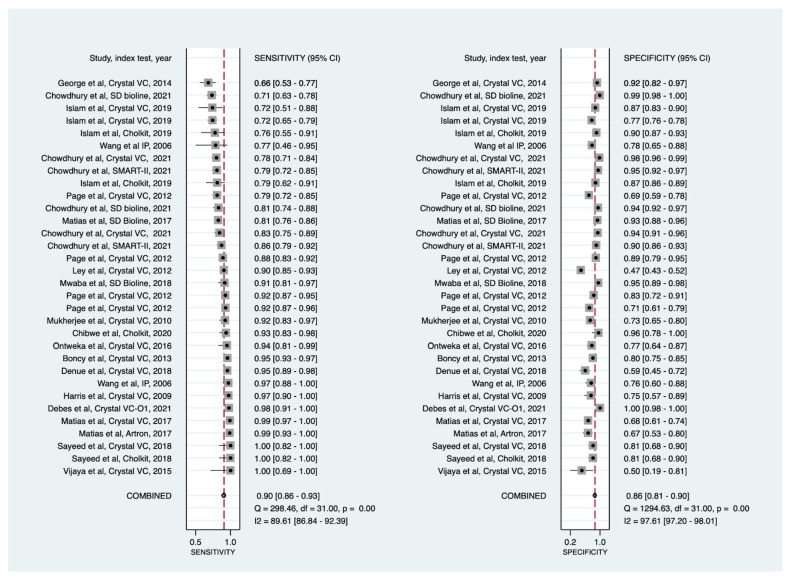
Forest plots of the sensitivities and specificities of cholera rapid diagnostic tests (direct stool testing) for the detection of *Vibrio cholerae O1*. CI = confidence interval; IP= Institut Pasteur. Data points are sorted by sensitivity performance.

**Figure 2:**
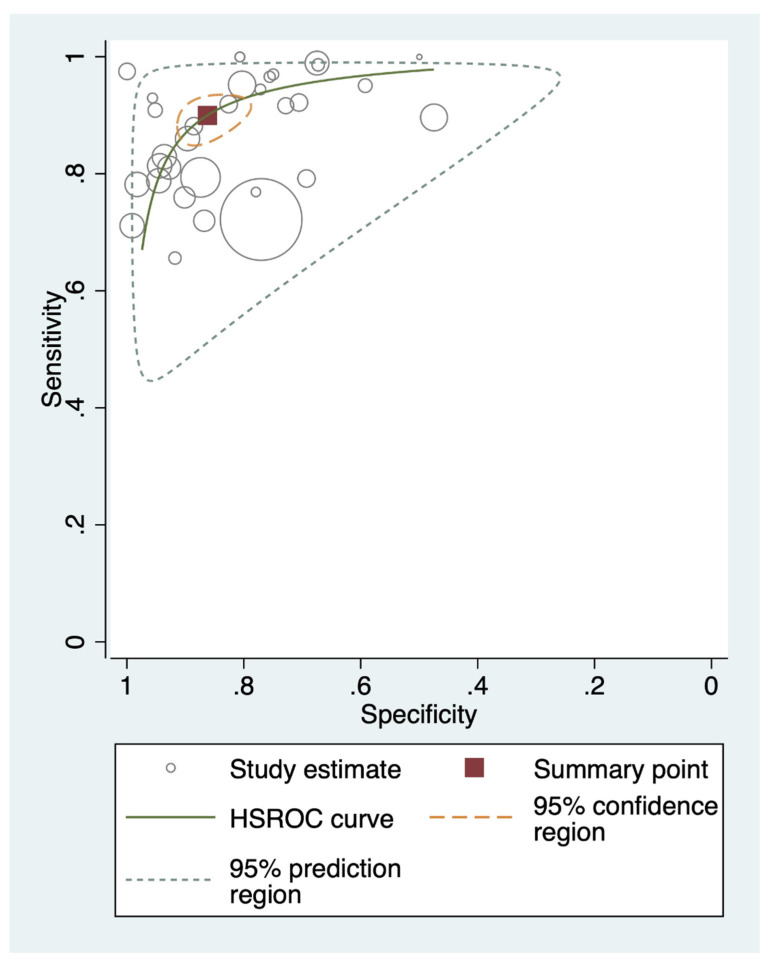
Hierarchical summary receiver-operating characteristic curves of the sensitivity and specificity of cholera rapid diagnostic tests (direct testing of fresh stools). Each circle represents the sensitivity and specificity of each included data point (*n* = 32). The summary point refers to pooled sensitivity and specificity. Sensitivity = 90% (95% CI, 86 to 93) and specificity = 86% (95% CI, 81 to 90). GTFCC recommends that cholera RDTs should be at least 90% sensitive and 85% specific. CI = confidence interval; HSROC = hierarchical summary receiver-operating characteristics; GTFCC = Global Task Force on Cholera Control.

Using the bivariate random-effects model (Table 2; Figure 1; and Supplementary Figure S2), direct specimen testing via cholera RDTs showed a pooled sensitivity of 90% (86% to 93%) and pooled specificity of 86% (81% to 90%) with moderate certainty of evidence (Table 3). The HSROC curve (Figure 2) shows greater heterogeneity in sensitivity (range: 66% to 100%) and specificity (range: 47% to 100%). About 47% of the data points (15/32) had specificity below 85%. Similarly, 47% of the data points (15/32) also had a sensitivity below 90%. The HSROC curve moderately approached the upper left-hand corner of the graph, indicating a moderate diagnostic performance.

#### 3.3.2. Sensitivity Analyses

The pooled sensitivity in the studies using direct specimens testing slightly decreased [88% (84% to 92%)] when we included three additional data points from the new study performed in India (where PCR was used as the gold standard) [14]. However, the pooled specificity slightly increased to 87% (83% to 91%) (Supplementary Figure S3A,B).

##### Crystal VC RDTs

**Figure 3:**
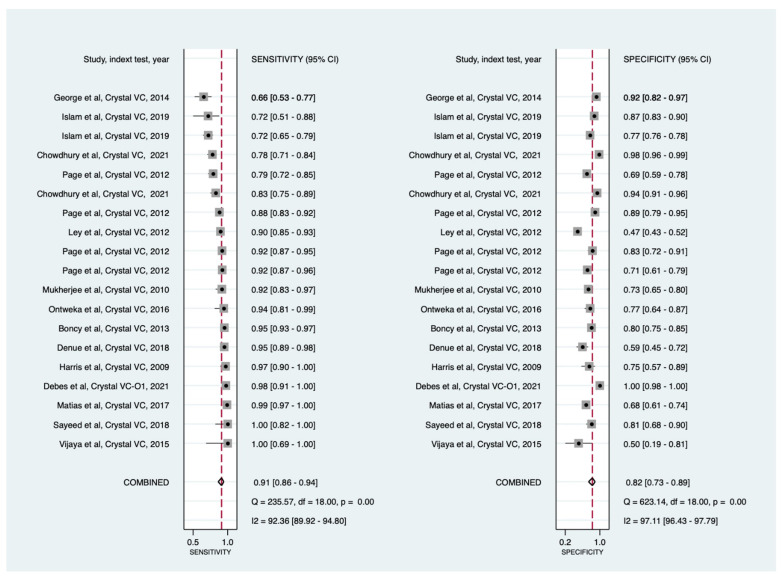
Forest plots of the sensitivities and specificities of Crystal VC cholera rapid diagnostic test for the detection of *Vibrio cholerae* O1 (direct stool testing).

When crystal VC was used for direct specimens testing (19 data points with 11,042 specimens), the pooled sensitivity and specificity were 91% (86% to 94%) and 82% (73% to 89%), respectively (Figure 3 and Supplementary Figure S4). Seven data points (37%; 7/19) had sensitivity estimates below the minimal performance of 90%, and only six data points (32%; 6/19) reached the minimal performance specificity of 85%.

It is important to note that one new study using Crystal VC-O1 reported higher estimates of sensitivity (98%) and specificity (100%) [13].

##### Cholera RDTs by Geographic Regions

**Figure 4:**
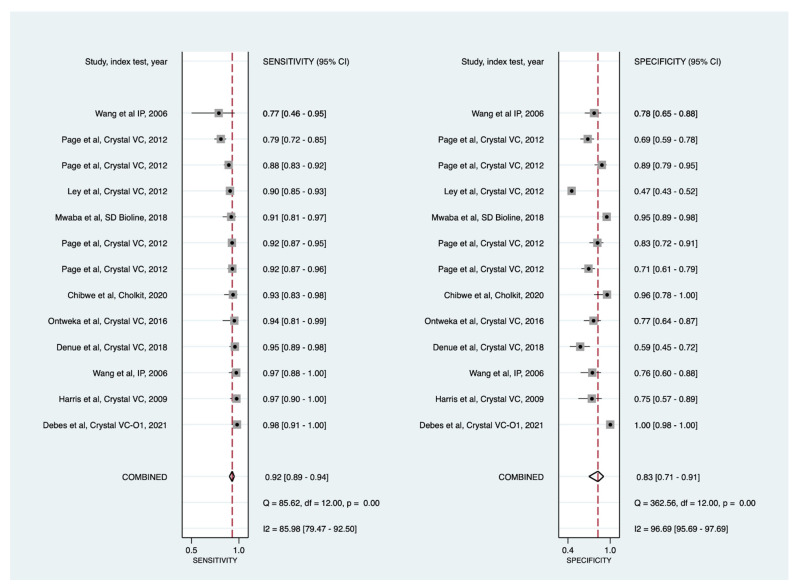
Forest plots of the sensitivities and specificities of cholera rapid diagnostic tests (direct stool testing) with their 95% confidence intervals. This subanalysis was restricted to studies conducted in Africa. CI = confidence interval; IP = Institut Pasteur.

**Figure 5:**
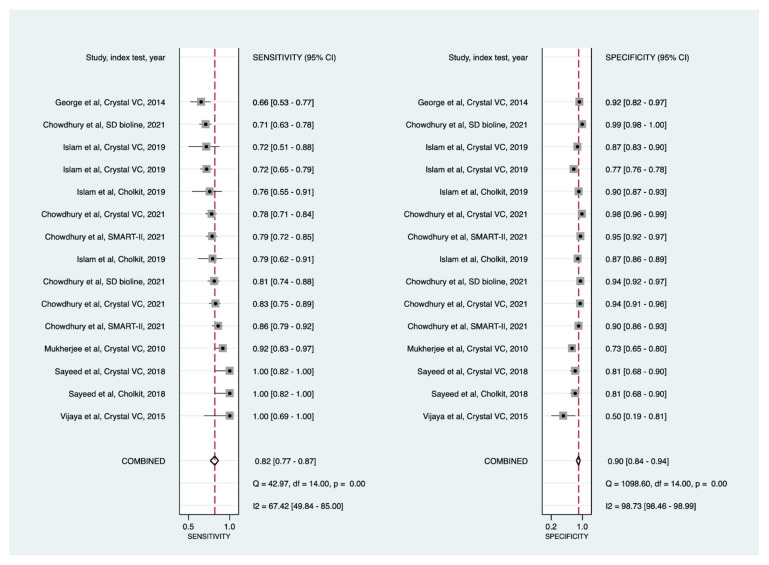
Forest plots of the sensitivities and specificities of cholera rapid diagnostic tests (direct stool testing) with their 95% confidence intervals. This sub-analysis was restricted to studies conducted in Asia (Bangladesh and India). CI = confidence interval.

**Figure 6:**
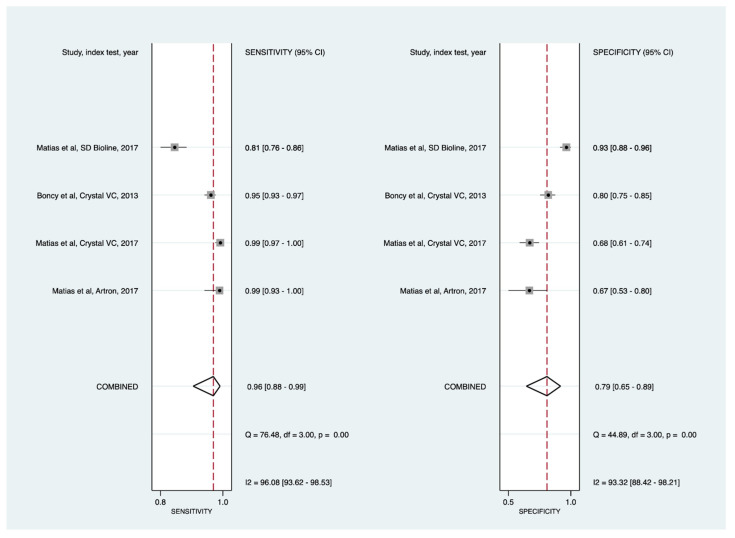
Forest plots of the sensitivities and specificities of cholera rapid diagnostic tests (direct stool testing) with their 95% confidence intervals. This subanalysis was restricted to studies conducted in the Americas (Haiti). CI = confidence interval.

We assessed whether cholera RDTs performance varied across settings. We noted that pooled sensitivity and specificity were highly variable when analyses were stratified by continents for direct specimens testing (Table 4; Figure 4, Figure 5 and Figure 6). Cholera Sensitivity was higher in studies conducted in Africa [92% (89% to 94%)] compared to those conducted in Asia [82% (77% to 87%)]. However, specificity [83% (71% to 91%)] was lower in Africa compared to Asia [90% (84% to 94%)]. Studies conducted in the Americas (Haiti) provided a pooled sensitivity of 96% (88% to 99%) and a pooled specificity of 79% (65% to 89%).

Outbreak-related stool specimens from Africa [16,19,21,24,25,27,32] and Haiti [23] showed sensitivity ≥90%.

##### Direct and APW Enrichment Testing

Pooled sensitivity was 90% (86% to 93%) and pooled specificity was 91% (87% to 94%) when all the 45 data points (with 19,280 specimens) from both direct stool testing and after APW enrichment were combined (Table 2 and Supplementary Figure S5).

## 4. Discussion

This updated meta-analysis assessed accuracy of RDTs used for cholera screening in suspected patients. We assessed the performance of current RDTs using 32 data points (for direct specimen testing) including eight new data points (25% of the included data points) identified since our previous meta-analysis [7]. Unlike our previous meta-analysis, in this meta-analysis, the outcome was restricted to the detection of *V. cholerae* O1 and the performance of cholera RDTs across continents was highlighted. The findings of current meta-analysis are consistent with those of our previous meta-analysis [7]: via direct specimen testing, cholera RDTs showed a moderate pooled sensitivity (90%; versus 91% in our previous meta-analysis) and specificity (86%; versus 80% in our previous meta-analysis). Study results were heterogenous with substantial uncertainty in performance; that is, the sensitivity (ranging from 66% to 100%) and specificity (ranging from 47% to 100%) of current cholera RDTs vary considerably, suggesting that improvements in the accuracy of cholera RDTs are urgently needed. We deduced some factors that may account for this heterogeneity with potential public health implications. For instance, location of RDTs usage was a source of heterogeneity in RDTs performance. RDTs showed a relatively higher pooled sensitivity but lower specificity in studies conducted in Africa and in the Americas than in Asia.

Although other explanations may be possible, we speculate that this relatively improved pooled sensitivity seen in Africa and the Americas could have been due to the fact that most of the stool specimens from Africa and the Americas were outbreak-related. However, the specimens from Asia were collected during surveillance. Since cholera RDTs were assessed during outbreaks in Africa, it was surmised that a significant number of samples were tested within a shorter period of time, which could have influenced RDT sensitivity. Furthermore, as *V. cholerae* strains may vary substantially in different geographical regions throughout the world, so too may the sensitivity of existing cholera RDTs. These data suggest that during surveillance or at the beginning of outbreaks, a negative cholera RDT result does not rule out cholera in a person with clinical symptoms of cholera. Thus, in such situations, even negative cholera RDT results should be confirmed using microbiological culture and/or PCR.

We noted that when the analyses were restricted to studies carried out in Africa, the specificity was 83%. The pooled specificity of 83% meant that for every 100 people tested who were not infected by *V. cholerae* O1, 17 had positive results. This is important in the context of concerns related to diverting the resources for unnecessary further testing (e.g., stool culture), which may increase the effort spent on testing. However, studies conducted in Asia showed slightly higher specificity (90%), which meant that 10% would have had a positive result without any infection. This suggests that every positive result obtained with cholera RDT does not automatically rule in cholera due to the potential for false positive results. During outbreaks, an increase of true positives is likely to be seen at the cost of increasing false positives. Cross-reactivity between *V. cholerae* O1 antibodies and “undefined entities” in stool specimens have been hypothesized to account for the false positives [26]. Issues occurring due to this suboptimal accuracy of current RDTs include the reluctance of health providers to report a cholera outbreak using RDT alone, or, conversely, to trigger an outbreak response using RDT alone. This, in turn, can delay a mitigatory response to a cholera outbreak [33].

For all these reasons, clinicians should be aware of the limitations of these RDTs (i.e., the unreliability of positive as well as negative results). Positive RDTs should always be validated by PCR, microbiological analyses, or the combination of both.

Despite their suboptimal accuracy, cholera RDTs remain a useful tool during outbreaks as they are suited to be used outside a laboratory setting, are easy to operate with a quick turnaround time, and are good for community health workers because they can help to detect cholera transmission in communities. In addition, the high accuracy of some newly developed cholera RDT brands such as Cholkit [16,17,18] and Crystal VC-O1 [13] suggests great potential and should be confirmed in more field studies. However, it should be noted that studies that evaluated Cholkit or Crystal VC-O1 were either industry-sponsored or received RDTs kits from the developer/manufacturer.

We could not perform meta-analysis on all RDTs on an individual basis due to a lack of data. Crystal-VC, the most tested RDT in the field, was assessed separately as new data points were available (Figure 3).

In this updated meta-analysis, Crystal VC pooled sensitivity was the same as in our previous estimate (91%), but pooled specificity increased to 82% (versus 75% in our previous estimates [7]). This slight increase in specificity is due, in part, to the high specificity found with the newly developed Crystal VC-O1 [13], and Crystal VC that was used with enriched culture methods by Chowdhury and colleagues [14]. This improved pooled specificity was still below the 85% specificity recommended by GTFCC [6]. Therefore, these data provide a unique opportunity to advocate for the continuation of research to develop and validate newer cholera RDTs.

It is crucial to remind health practitioners that the selection of a gold standard may impact the sensitivity and specificity of an index test. Simply, an imperfect gold standard bias has raised concerns about underestimating the sensitivity and specificity of an index test [34]. Microbiological culture can be affected by viable but non-culturable *V. cholerae*, antibiotics consumption, and lytic bacteriophages [35,36]. For instance, one study reported the presence of *V. cholerae* O1 lytic phages (denoting cholera etiology) in half of the dipstick tests that were positive for *V. cholerae* O1, but those stool specimens were negative using culture [36]. Some misclassification with PCR may occur: PCR can misclassify a patient without cholera as having the disease if the *V. cholerae* cells are dead or the quantity of viable cells is low in a stool sample, especially in the context of the prior administration of antibiotics [26]. Therefore, it is theoretically possible that in studies where PCR was used as the gold standard, the sensitivity of cholera RDTs would have been underestimated. In this meta-analysis, of all the studies reviewed concerning direct testing, three studies used PCR as the gold standard: sensitivity was reportedly high in two studies, between 94% [32] and 97% [27]. However, sensitivity was low when cholera RDTs were compared with PCR in one study, between 52% and 58% [14], which affected the overall RDTs pooled sensitivity [i.e., slightly decreased to 88% (Supplementary Figure S3A,B)]. Poor laboratory capacity can make the matter worse. For example, during an outbreak in Nigeria, a local laboratory failed to confirm a positive RDT by microbiological culture (provided negative result by stool culture), but a positive RDT was subsequently confirmed in a regional reference laboratory ten days later [33].

A notable input of this current review is the use of the GRADE approach (Table 3). We found that certainty of evidence was moderate, driven in large part by the potential for bias associated with difficulties to ascertain methods for selecting or excluding patients in some studies, inconsistency (considerable variability in sensitivity and specificity across studies), and imprecision (most of the studies reported wider 95% CI).

One of the limitations of this study stems from our inability to account for disease severity effect in this meta-analysis because of a lack of data. It is, therefore, imperative for future field studies to evaluate the performance of cholera RDTs considering the disease severity. As a case in point, one study reported that cholera RDTs performance were similar across disease spectrum [14].

It is important to note that existing commercially available RDTs for infectious diseases vary widely in sensitivity and specificity performance, depending on the RDT brand and ailment. D. Bouzid and colleagues have recently summarized their reliability and validity in clinical settings [37].

We conclude that current cholera RDTs have moderate accuracy. Cholera RDTs will continue to be helpful in outbreak detection or surveillance purposes, ultimately assisting in cholera control efforts. It is therefore crucial for primary health practitioners to be aware of their availability, their performance, and limitations. These data call for research to develop alternative, simple cholera RDTs with both high sensitivity and specificity. In addition, more field evaluation on the performance of Cholkit and Crystal VC-O1 is needed.

## Figures and Tables

**Table 1:** Characteristics of included studies.

Study Characteristic		Studies of Cholera Rapid Tests (N = 20), *n* (%)
Study design	Cross-sectional	20 (100)
Industry funded	Yes	2 (10.0)
	No	11 (55.5)
	Not reported	7 (35.5)
Specimen type	Stool	20 (100)
Testing type	Direct stool testing	11 (55.0)
	Stool enrichment with alkaline peptone water	3 (15.0)
	Both	6 (30.0)
Commercial brand	Crystal VC	15 (75.0)
	Crystal VC-O1	1 (5.0)
	Cholkit	3 (15.0)
	Pasteur Cholera Dipstick	3 (15.0)
	SD Bioline	3 (15.0)
	Cholera Smart O1	1 (5.0)
	Smart II Cholera O1	1 (5.0)
*Vibrio cholera* strain detected	*Vibrio cholera* O1	20 (100)
Setting	Africa	11 (55.5)
	Asia	7 (35.0)
	Americas	2 (10.0)

**Table 2:** Results of pooled sensitivity and specificity of cholera RDTs using direct fresh stool for *V. cholerae* O1 detection.

Test	Data Point (*n*)	Sample Size (*n*)	Pooled Sensitivity (95% CI), %	Pooled Specificity (95% CI), %	Positive LR (95% CI)	Negative LR (95% CI)	DOR (95% CI)
All *	45	19,280	90 (86 to 93)	91 (87 to 94)	10 (7 to 15)	0.11 (0.08 to 0.15)	89 (56 to 142)
Direct fresh stool	32	15,877	90 (86 to 93)	86 (81 to 90)	7 (5 to 9)	0.12 (0.09 to 0.16)	56 (37 to 86)

Definition of abbreviations: RDT = rapid diagnostic test; CI = confidence interval; LR = likelihood ratio; DOR = diagnostic odds ratio; APW = alkaline peptone water. * All (we included all data points: direct stool testing and after APW enrichment). Included tests were: Crystal VC; Cholkit; Institut Pasteur cholera dipstick; SD Bioline; Smart; SMART-II; Crystal VC-O1; and Artron.

**Table 3:** GRADE certainty of evidence for cholera RDTs: should current cholera RDTs be used in patients suspected of cholera for surveillance or earlier outbreak detection?

Outcome	Number of Studies (Number of Specimens)	Study Design	Factors that May Lower Certainty of Evidence					Test Accuracy Certainty of Evidence
			Risk of bias	Indirectness	Inconsistency	Imprecision	Publication bias	
True positives (patients correctly identified as having with cholera)	20 (15,877)	Cross-sectional	Serious ^a^	Not serious	Very serious ^b^	Serious ^c^	Likely ^d^	Moderate ⊕⊕⊕
False negative (patients incorrectly identified as not having cholera)	20 (15,877)	Cross-sectional	Serious ^a^	Not serious	Very serious ^b^	Serious ^c^	Likely ^d^	Moderate ⊕⊕⊕ 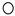
True negatives (patients correctly identified as not having cholera)	20 (15,877)	Cross-sectional	Serious ^a^	Not serious	Very serious ^b^	Serious ^c^	Likely ^d^	Moderate ⊕⊕⊕ 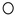
False positives (patients incorrectly identified as having cholera)	20 (15,877)	Cross-sectional	Serious ^a^	Not serious	Very serious ^b^	Serious ^c^	Likely ^d^	Moderate ⊕⊕⊕ 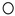

**^a^** Methods for selecting patients were difficult to ascertain in some studies. ^b^ There was heterogeneity in study results: sensitivity and specificity varied across cholera rapid test brands. ^c^ Many data points generated wider 95% confidence intervals. ^d^ We assumed some degree of publication bias because studies in which cholera rapid diagnostic tests had poor performance were probably less likely to be published. However, we did not downgrade the quality of evidence as a formal assessment of publication bias was not performed.

**Table 4:** Pooled sensitivity and specificity of cholera RDTs stratified by geographical regions.

Subgroup	Data Point (*n*)	Sample Size (*n*)	Pooled Sensitivity (95% CI), %	Pooled Specificity (95% CI), %	Positive LR (95% CI)	Negative LR (95% CI)	DOR (95% CI)
Africa *	13	2644	92 (89 to 94)	83 (71 to 91)	6 (3 to 10)	0.09 (0.06 to 0.14)	59 (24 to 145)
Asia (Bangladesh and India) **	15	11,527	82 (77 to 87)	90 (84 to 94)	8 (5 to 13)	0.20 (0.15 to 0.26)	42 (26 to 69)
Americas (Haiti) ***	4	1706	96 (88 to 99)	79 (65 to 89)	5 (3 to 6)	0.05 (0.02 to 0.13)	99 (52 to 187)

Definition of abbreviations: RDT = rapid diagnostic test; CI = confidence interval; LR = likelihood ratio; DOR = diagnostic odds ratio. * Included tests were: Crystal VC; Cholkit; Institut Pasteur cholera dipstick; SD Bioline; Smart; and Crystal VC-O1. ** Included tests were: Crystal VC; Cholkit; SD Bioline; Smart; and SMART-II. *** Included tests were: Crystal VC; SD Bioline; and Artron.

## Supplementary Materials

The following are available online at https://www.mdpi.com/article/10.3390/diagnostics11112095/s1, Figure S1: Flow chart of included studies, Figure S2: Forest plots of the sensitivities and specificities of cholera rapid diagnostic tests (direct stool testing) for the detection of *Vibrio cholerae* O1, Figure S3: (A) Hierarchical summary receiver-operating characteristic curves of the sensitivity and specificity of cholera rapid diagnostic tests (direct stool testing), (B) Forest plots of the sensitivities and specificities of cholera rapid diagnostic tests, Figure S4: Forest plots of the sensitivities and specificities of Crystal VC cholera rapid diagnostic test for the detection of *Vibrio cholerae* O1 (direct stool testing), Figure S5: Hierarchical summary receiver-operating characteristic curves of the sensitivity and specificity of cholera rapid diagnostic tests (direct testing stools and after alkaline peptone water enrichment).

## Appendix A

**Table A1:** Summary of reviewed studies.

Study	Location	Study Period	Study Design	Participants’ Age (Year; Mean or Median)/Descriptor	User	Industry Funded (Yes or No)	Population	Specimen Type	Index Test	Reference Standard	Sample Size
Debes et al., 2021 [13]	Kenya	2018 to 2019	Cross-sectional	All age groups (mean = 25)	Lab technician	No, but received RDT kit from the manufacturer	Hospital samples: Individuals presenting to a health facility with acute watery diarrhea.	Stool	Crystal VC-O1	Culture and PCR	230
Chowdhury et al., 2021 [14]	India	2016 and 2017	Cross-sectional	All age groups	Lab technician	No	Hospital samples: Individuals hospitalized for diarrhea and children treated for diarrhea as outpatients at designated hospitals.	Stool	SD bioline cholera, SMART-II Cholera O1 and Crystal VC	Culture and PCR	506
Chibwe et al., 2020 [16]	Malawi	2018	Cross-sectional	All age groups (<5:14%; and >5: 86%)	Lab technician	No, but received RDT kit as a gift	Hospital samples: Individuals presenting to cholera treatment camps with acute diarrhea.	Stool (bulk stool or rectal swabs)	Cholkit	Culture	80
Islam et al., 2019 [17]	Bangladesh	Ongoing surveillance since 2016	Cross-sectional	Mean = 19	Lab technician	Yes	Hospital samples: Individuals presenting to hospitals with acute watery diarrhea.	Stool	Crystal VC	Culture	381
											5865
											614
								Stool	Cholkit	Culture	381
											1355
											424
Mwaba et al., 2018 [19]	Zambia	2016	Cross-sectional	Mean = 24	Lab technician	Not reported	Hospital samples: Patients with acute non-blood watery diarrhea.	Stool	SD Bioline cholera	Culture	170
Denue, 2018 [21]	Nigeria	2017	Cross-sectional	Mean = 20	Lab technician	No	Hospital samples: Individuals presenting to a cholera treatment unit with diarrhea.	Stool	Crystal VC	Culture	156
Sayeed et al., 2018 [18]	Bangladesh	Not reported	Cross-sectional	Median = 26	Lab technician	Yes	Hospital samples: Patients presenting to the icddr, b hospital with acute watery diarrhea.	Stool	Cholkit	Culture	76
								Stool	Crystal VC		76
Matias et al., 2017 [31]	Haiti	2014–2015	Cross-sectional	Not reported	Lab technician	No	Hospital samples: Patients presenting to a cholera treatment center with acute watery diarrhea.	Stool	Crystal VC	Culture	511
									Artron	Culture	129
									SD Bioline	Culture	451
Bwire et al., 2017 [30]	Uganda	2015	Cross-sectional	All age groups	Lab technician	No	Hospital samples: Suspected cholera patients presenting to hospitals.	Stool/rectal swabs	Crystal VC	Culture	102
Ontweka et al., 2016 [32]	South Sudan	2015	Cross-sectional	Median = 26	Lab technician	No	Hospital samples: Patients presenting to cholera treatment centers with acute watery diarrhea.	Stool	Crystal VC	PCR	101
Debes et al., 2016 [20]	Cameroon	2013–2014	Cross-sectional	All age groups	Lab technician	No	Hospital samples: Patients with acute non-blood watery diarrhea.	Stool	Crystal VC	PCR	673
Vijaya et al., 2015 [15]	India	Not reported	Cross-sectional	Not reported	Researcher	Not reported	Hospital samples: Patients presenting to a tertiary care hospital with acute watery diarrhea	Stool (18 bulk stool samples and 2 rectal swabs)	Crystal VC	Culture	20
George et al., 2014 [22]	Bangladesh	2013	Cross-sectional	Median = 32	Lab technician	Not reported	Hospital samples: Patients presenting to the icddr, b hospital with moderate to severe dehydration and acute watery diarrhea.	Stool	Crystal VC	Culture	125
Boncy et al., 2013 [23]	Haiti	2011	Cross-sectional	Not reported	Lab technician	Not reported	Hospital samples: Patients with acute rice watery diarrhea.	Stool	Crystal VC	Culture	644
Ley et al., 2012 [24]	Tanzania	2009	Cross-sectional	Not reported	Lab technician/Field workers	No	Hospital samples: Patients presenting to treatment centers with watery diarrhea.	Stool	Crystal VC	Culture	622
Page et al., 2012 [25]	DR Congo	2008	Cross-sectional	>5	Lab technician/Field worker	No	Hospital samples: Patients presenting to cholera treatment centers with acute watery diarrhea.	Stool	Crystal VC	Culture	256
										Culture and/or PCR	256
Mukherjee et al., 2010 [26]	India	2008	Cross-sectional	All age groups	Lab technician	Not reported	Hospital samples: Hospitalized patients with diarrhea.	Stool	Crystal VC	Culture	212
Harris et al., 2009 [27]	Guinea-Bissau	2008	Cross-sectional	Median = 27	Lab technician	Not reported	Hospital samples: Patients presenting to a hospital cholera ward.	Stool	Crystal VC	PCR	101
Wang et al., 2006 [28]	Mozambique	2004	Cross-sectional	Mean = 20 (cholera) and 24 (non-cholera)	Lab technician	No	Hospital samples: Patients with acute non-blood watery diarrhea.	Bulk stool	Institut Pasteur cholera dipstick	Culture	172
								Rectal swabs	Institut Pasteur cholera dipstick	Culture	219
Bhuiyan et al., 2003 [29]	Bangladesh	2002	Cross-sectional	24	Lab technician	Not reported	Hospital samples: Patients hospitalized at the icddr, b for diarrhea.	Rectal swabs	Institut Pasteur cholera dipstick	Culture	134

Definition of abbreviations: RDT = rapid diagnostic test; PCR = polymerase chain reaction, icddr, b = International Centre for Diarrhoeal Disease Research, Bangladesh.

**Table A2:** Raw data extracted from studies included in the meta-analysis.

Study	Index Test	Sample Tested	Direct Specimen or after Enrichment	Results, *n*			
				True Positive	False Positive	False Negative	True Negative
Debes et al., 2021 [13]	Crystal VC-O1	Stool	Direct	79	0	2	149
Chowdhury et al., 2021 [14]	SD Bioline	Stool	Direct *	115	11	105	275
	SMART-II	Stool	Direct *	128	22	92	264
	Crystal VC	Stool	Direct *	122	9	98	277
Chowdhury et al., 2021 [14]	SD Bioline	Stool	Direct	105	21	24	356
			Direct **	111	3	45	347
	SMART-II	Stool	Direct	111	39	18	338
			Direct **	123	19	33	331
	Crystal VC	Stool	Direct	107	24	22	353
			Direct **	122	6	34	344
Chibwe et al., 2020 [16]	Cholkit	Stool	Direct	53	1	4	22
			Enrichment	56	0	1	23
Islam et al., 2019 [17]	Crystal VC	Stool	Direct	18	47	7	309
			Direct	117	1308	45	4395
			Enrichment	17	9	8	347
			Enrichment	28	53	13	520
	Cholkit	Stool	Direct	19	35	6	321
			Direct	27	166	7	1155
			Enrichment	16	21	9	335
			Enrichment	20	22	10	372
Mwaba et al., 2018 [19]	SD Bioline	Stool	Direct	60	5	6	99
			Enrichment	63	0	3	104
Denue, 2018 [21]	Crystal VC	Stool	Direct	97	22	5	32
Sayeed et al., 2018 [18]	Cholkit	Stool	Direct	19	11	2	44
	Crystal VC	Stool	Direct	19	11	2	44
Matias et al., 2017 [31]	Crystal VC	Stool	Direct	282	65	3	135
	SD Bioline	Stool	Direct	197	15	46	193
	Artron	Stool	Direct	73	17	1	35
Bwire et al., 2017 [30]	Crystal VC	Stool/Rectal swabs	Enrichment	91	1	1	9
Ontweka et al., 2016 [32]	Crystal VC	Stool	Direct	34	13	2	44
			Enrichment	31	0	5	64
Debes et al., 2016 [20]	Crystal VC	Stool	Enrichment	25	3	7	638
Vijaya et al., 2015 [15]	Crystal VC	Stool	Direct **	10	5	0	5
George et al., 2014 [22]	Crystal VC	Stool	Direct	42	5	22	56
			Enrichment	48	1	16	60
Boncy et al., 2013 [23]	Crystal VC	Stool	Direct	381	48	19	196
Ley et al., 2012 [24]	Crystal VC	Stool	Direct	182	220	21	199
Page et al., 2012 [25]	Crystal VC	Stool	Direct	142	30	12	72
			Direct	122	31	32	70
			Direct	164	8	22	62
			Direct	171	12	15	57
Mukherjee et al., 2010 [26]	Crystal VC	Stool	Direct	66	38	6	102
Harris et al., 2009 [27]	Crystal VC	Stool	Direct	65	8	2	24
Wang et al., 2006 [28]	Institut Pasteur cholera dipstick	Stool/Rectal swabs	Direct	57	10	2	31
			Direct	10	13	3	46
			Enrichment	45	3	0	40
			Enrichment	19	1	2	109
Bhuiyan et al., 2003 [29]	Institut Pasteur cholera dipstick	Rectal swabs	Enrichment	65	5	3	61

* Polymerase chain reaction used as gold standard. ** Enriched culture used as gold standard.

**Table A3:** QUADAS-2 assessments.

Study	Risk of Bias				Applicability Concerns		
	Patient Selection	Index Test	Reference Standard	Flow and Timing	Patient Selection	Index Test	Reference Standard
Debes et al., 2021 [13]							
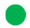
Chowdhury et al., 2021 [14]							
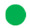
Islam et al., 2019 [17]							
Mwaba et al., 2018 [19]							
Denue BA, 2018 [21]							
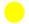
Chibwe et al., 2020 [16]							
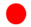
Sayeed et al., 2018 [18]							
Matias et al., 2017 [31]							
Bwire et al., 2017 [30]							
Ontweka et al., 2016 [32]							
Debes et al., 2016 [20]							
Vijaya et al., 2015 [15]							
George et al., 2014 [22]							
Boncy et al., 2013							
Ley et al., 2012							
Page et al., 2012 [25]							
Mukherjee et al., 2010 [26]							
Harris et al., 2009 [27]							
Wang et al., 2006 [28]							
Bhuiyan et al., 2003 [29]							

Green represents low risk of bias, yellow represents unclear risk of bias, and red represents high risk of bias.
