# Supplementary material for: Cholera Rapid Diagnostic Tests for the Detection of Vibrio cholerae O1: An Updated Meta-Analysis
Source: Diagnostics (Basel). 2021 Nov 13;11(11):2095. doi: 10.3390/diagnostics11112095 (PMC8622830; doi:10.3390/diagnostics11112095)

Supplementary Files

Supplementary Figure S1. Flow chart of included studies

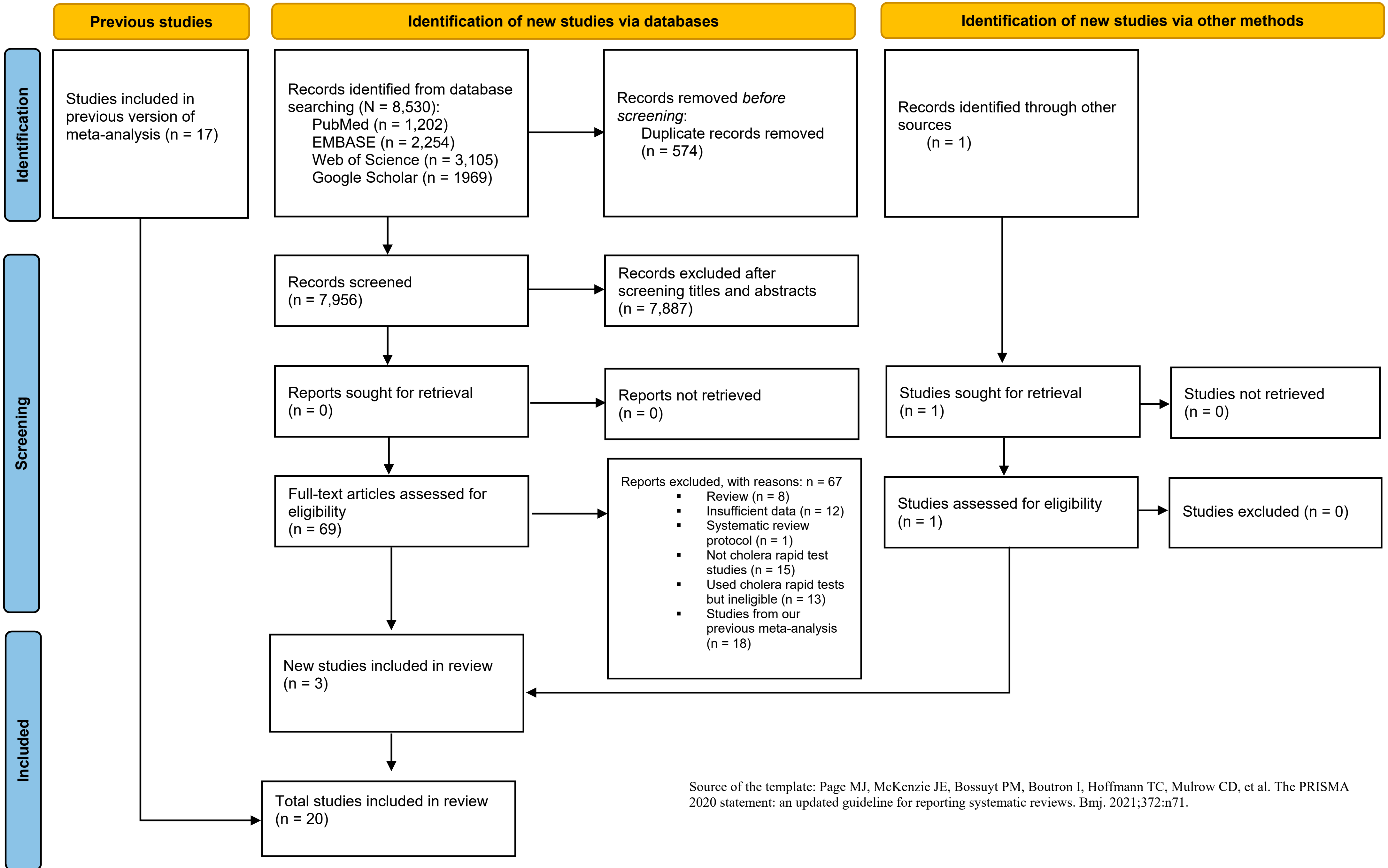

Source of the template: Page MJ, McKenzie JE, Bossuyt PM, Boutron I, Hoffmann TC, Mulrow CD, et al. The PRISMA 2020 statement: an updated guideline for reporting systematic reviews. Bmj. 2021;372:n71.

Supplementary Figure S2. Forest plots of the sensitivities and specificities of cholera rapid diagnostic tests (direct stool testing) for the detection of *Vibrio cholerae O1*. CI=confidence interval; IP= Institut Pasteur. Data points are sorted by specificity performance.

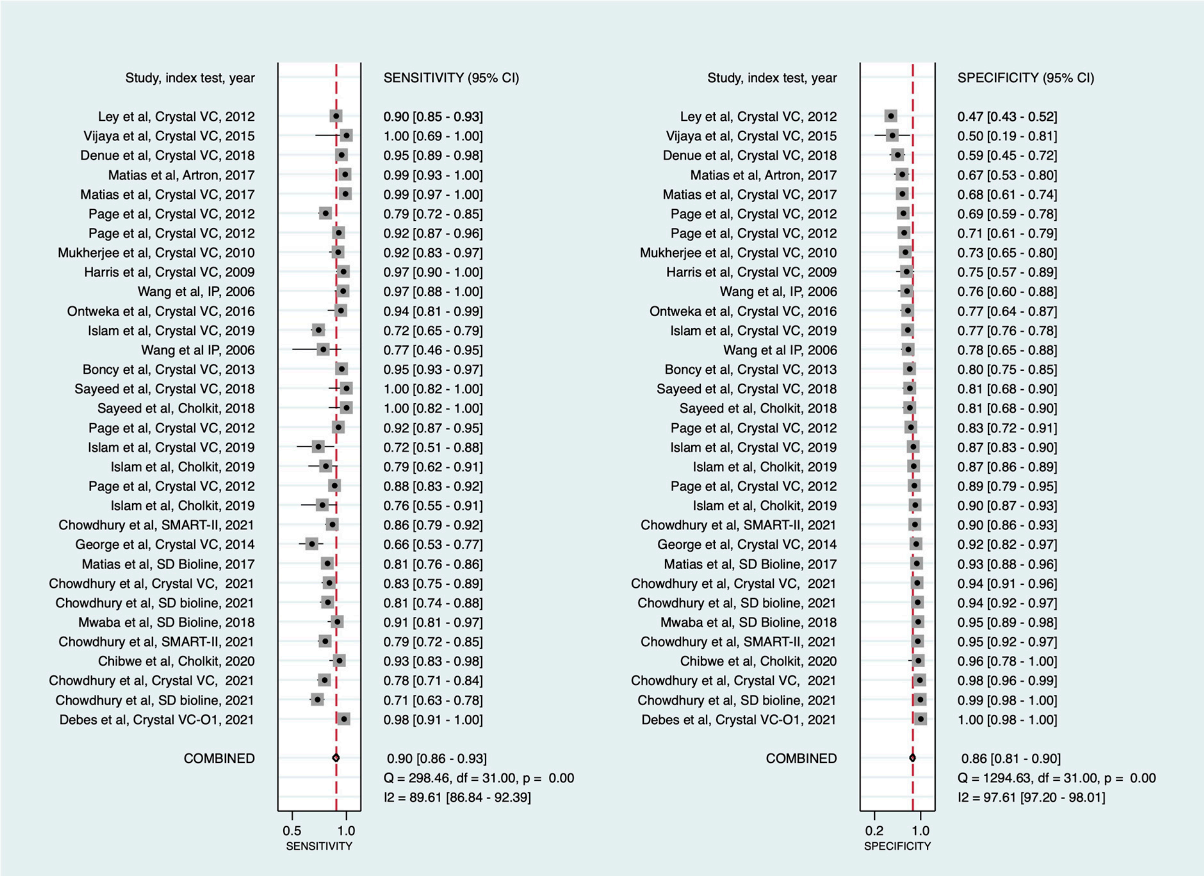

Supplementary Figure S3.

3A. Hierarchical summary receiver-operating characteristic curves of the sensitivity and specificity of cholera rapid diagnostic tests (direct stool testing). Each circle represents the sensitivity and specificity of each included data point (n= 35 with 17,395 specimens). The summary point refers to pooled sensitivity and specificity. Sensitivity = 88% (95% CI, 84% to 92%) and specificity = 87% (95% CI, 83% to 91%). CI = confidence interval; HSROC= hierarchical summary receiver-operating characteristics.

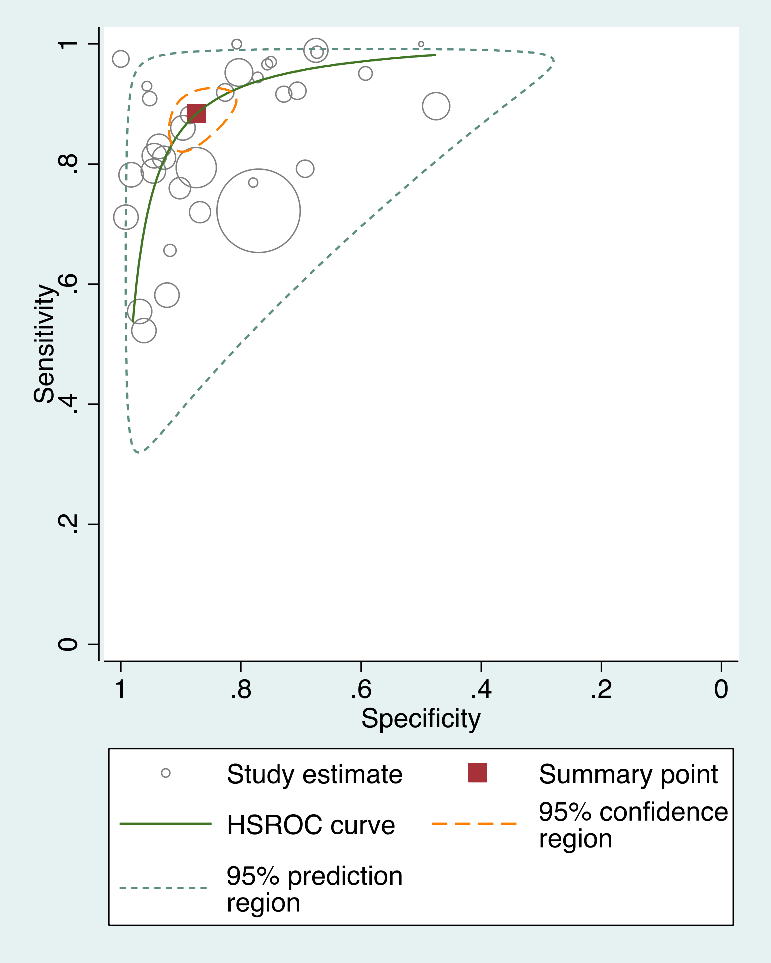

3B. Forest plots of the sensitivities and specificities of cholera rapid diagnostic tests (direct stool testing; n= 35 with 17,395 specimens) for the detection of *Vibrio cholerae O1*. CI=confidence interval; IP= Institut Pasteur.

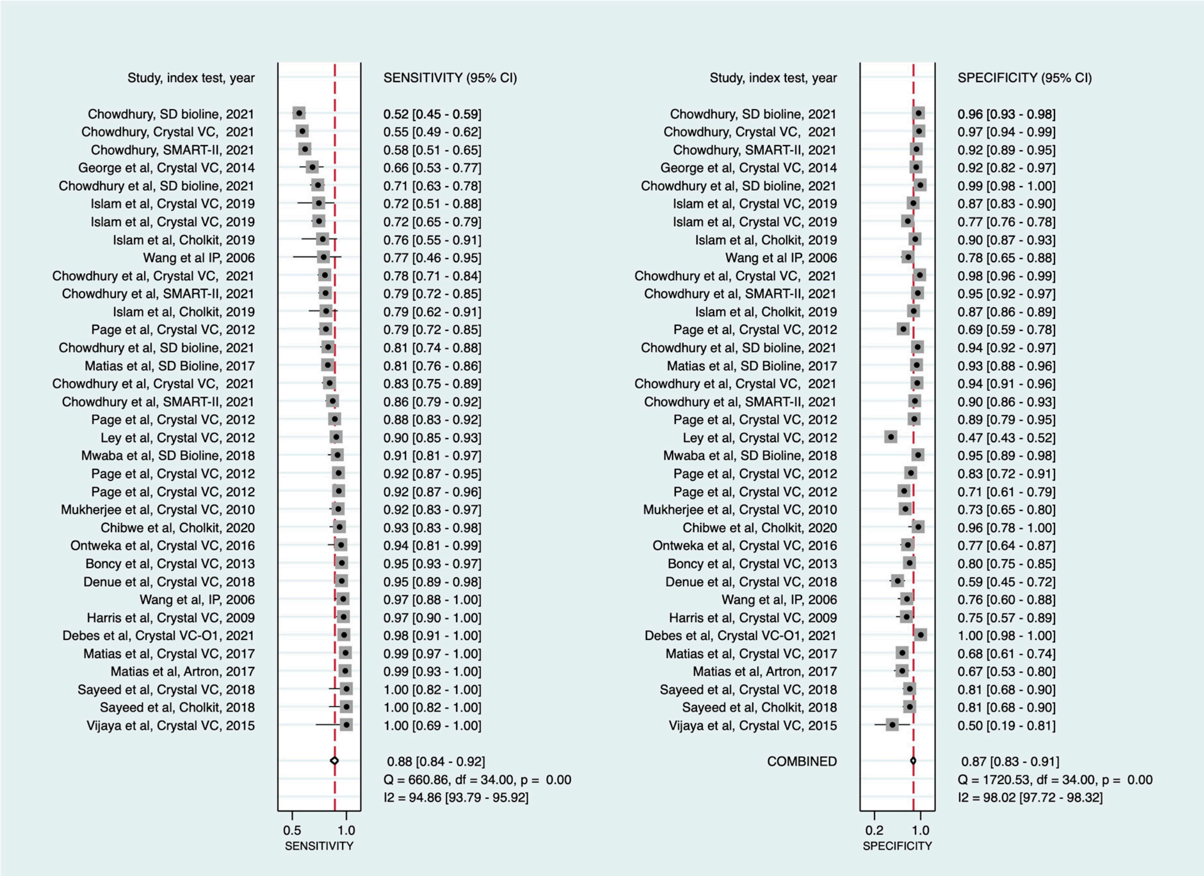

Supplementary Figure S4. Forest plots of the sensitivities and specificities of Crystal VC cholera rapid diagnostic test for the detection of *Vibrio cholerae O1* (direct stool testing). Data points are sorted by specificity performance.

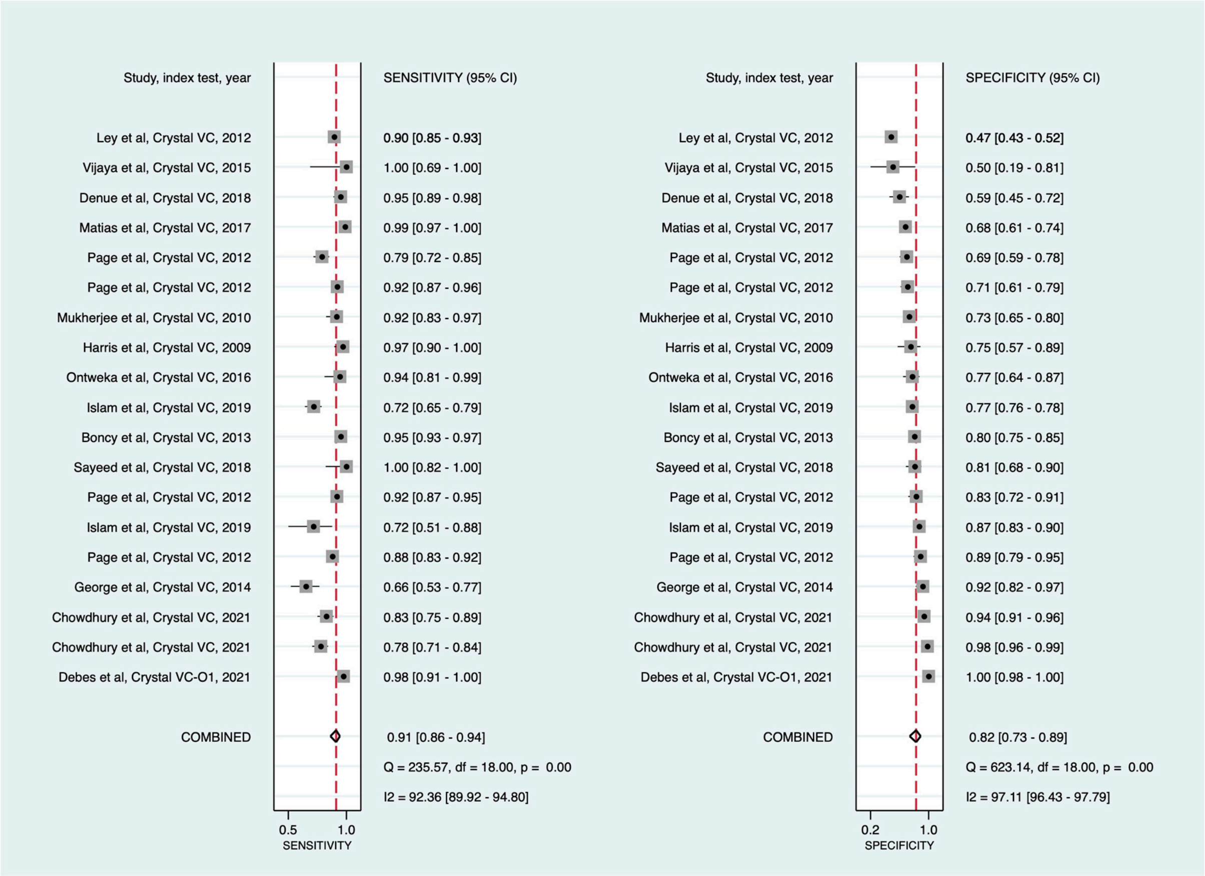

Supplementary Figure S5.

Hierarchical summary receiver-operating characteristic curves of the sensitivity and specificity of cholera rapid diagnostic tests (direct testing stools and after alkaline peptone water enrichment). Each circle represents the sensitivity and specificity of each included data point (n= 45 with 19,280 specimens). The summary point refers to pooled sensitivity and specificity. Sensitivity = 90% (95% CI, 86% to 93%) and specificity = 91% (95% CI, 87% to 94%). CI = confidence interval; HSROC= hierarchical summary receiver-operating characteristics.

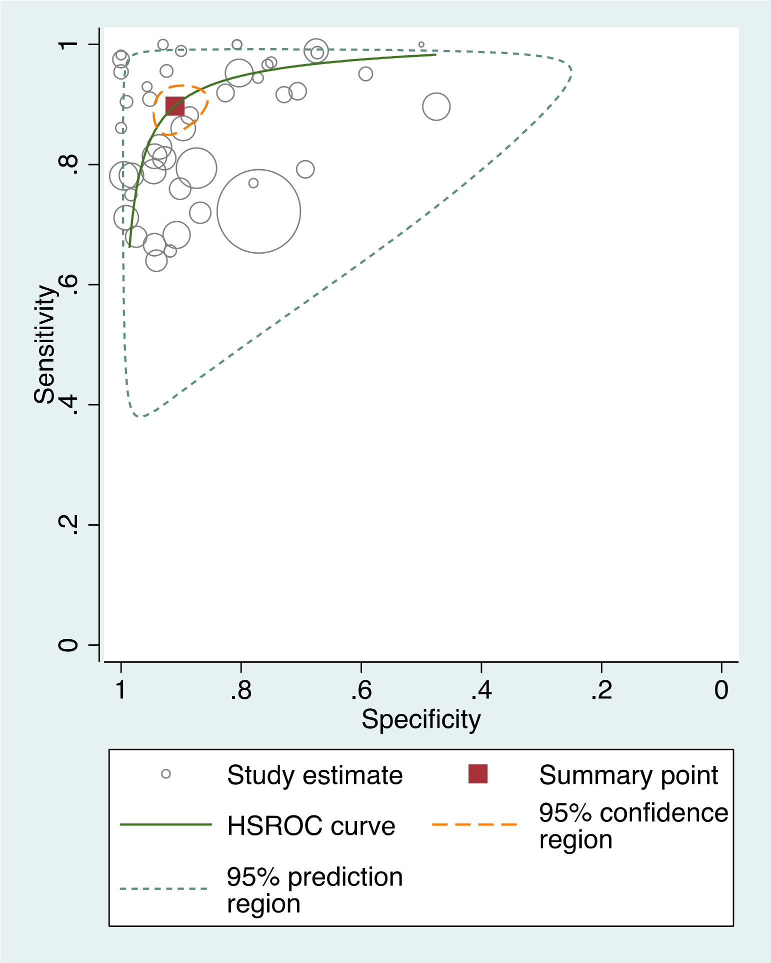

Supplement: Supplementary file 1 [file diagnostics-11-02095-s001.zip › diagnostics-1440057-supplementary.pdf]
